# Development and Validation of an HPLC Method for Simultaneous Determination of Capsaicinoids and Camphor in Over-the-Counter Medication for Topical Use

**DOI:** 10.3390/molecules27041261

**Published:** 2022-02-14

**Authors:** Martin Lalić, Ana Soldić, Andrija Lalić, Zdenka Lalić, Miranda Sertić

**Affiliations:** 1 Apipharma d.o.o., Pharmaceutical-Food, Cosmetic Industry and Trade, Jeronima Kavanjina 26, 10090 Zagreb, Croatia; martin@apipharma.hr (M.L.); analytical@apipharma.hr (A.S.); andrija@apipharma.hr (A.L.); zdenka@apipharma.hr (Z.L.); 2 University of Zagreb, Faculty of Pharmacy and Biochemistry, Department of Pharmaceutical Analysis, A. Kovačića 1, 10000 Zagreb, Croatia

**Keywords:** capsaicin, dihydrocapsaicin, camphor, HPLC, validation, OTC

## Abstract

A reverse-phase high-performance liquid chromatography method was developed to determine and quantify capsaicin (*trans*-8-methyl-N-vanillyl-6- nonenamid), dihydrocapsaicin (8-methyl-N-vanillylnonanamide), and camphor (trimethylbicyclo[2.2.1]heptan-2-one). It is applicable in analyses of over-the-counter (OTC) medications for topical use and raw materials such as chili pepper oleoresin. Chromatographic separation was carried out on a C18 column using an isocratic flow of the mobile phase containing acetonitrile and ultrapure water in a ratio of 2:3, with pH adjusted to 3.2 using glacial acetic acid, and a flow rate of 1.5 mL/min. The concentration of the eluting compounds was monitored by a diode-array detector at a wavelength of 281 nm. The method was evaluated for several validation parameters, including selectivity, accuracy (confidence intervals < 0.05%), repeatability, and intermediate precision. The limit of detection (LOD) was determined to be 0.070 µg/mL for capsaicin, 0.211 µg/mL for dihydrocapsaicin, and 0.060 µg/mL for camphor. The limit of quantification (LOQ) was determined to be 0.212 µg/mL for capsaicin, 0.640 µg/mL for dihydrocapsaicin, and 0.320 µg/mL for camphor. Linearity was set in the range of 2.5–200 µg/mL for capsaicin and dihydrocapsaicin and 25–2000 µg/mL for camphor. The suggested analytical method can be used for quality control of formulated pharmaceutical products containing capsaicinoids, camphor, and propolis.

## 1. Introduction

**Figure 1:**
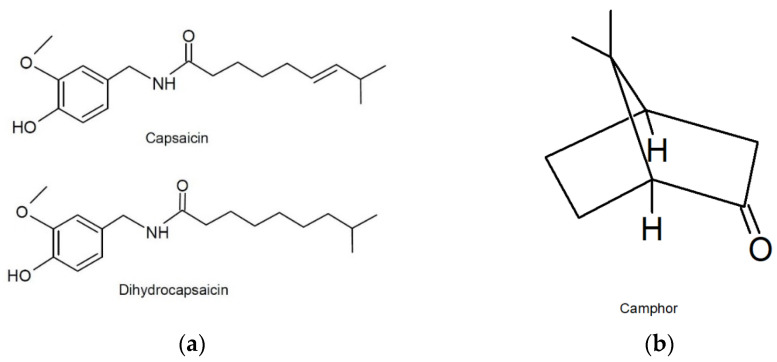
Structural formulas of (**a**) capsaicin, dihydrocapsaicin, and (**b**) camphor.

Capsaicinoids are a group of unique molecules characteristic of chili peppers [1]. According to their chemical structure (Figure 1), they are amides of vanillylamine and carboxylic acids. Capsaicin (*trans*-8-methyl-N-vanilyl-6-nonenamide), along with dihydrocapsaicin and nordihydrocapsaicin, is the most common in the genus of pepper (*Capsicum annum*), from which it is most usually extracted for use in the pharmaceutical industry. Capsaicin has proven biological effects. It has analgesic and anti-inflammatory effects by agonist action on type 1 vanilloid receptors, TRPV1 receptors, (transient receptor potential vanilloid type-1). TRPV1 is a nonselective cation channel with high Ca^2+^ permeability. When activated, the channel can transiently open and initiate depolarization due to the influx of calcium and sodium ions. By binding to them, inflammation in the application area is reduced, and sensory neurons are desensitized, resulting in a rapid analgesic effect [2,3,4]. Because of these two effects, capsaicin is used in various over-the-counter (OTC) pharmaceutical formulations for topical administration, especially for ailments that accompany rheumatism, arthritis, and various muscle injuries. Morgan Hall et al. [5] showed that capsaicin could be a good treatment for neuropathic pain and neuropathic pain-related disease states due to fewer side effects when compared with opioids, gabapentin, tricyclic antidepressants, and serotonin-norepinephrine reuptake inhibitors that are currently used. Besides analgesic and anti-inflammatory effects, capsaicin shows a strong enhancing effect on the expression of thermogenic proteins, which leads to browning of white adipose tissue and activation of brown adipose tissue [6]. Capsaicinoids are used in different concentrations in the forms of liquid formulation (concentrations of 10–20%), patches (concentration of 8%), gel (concentrations of 2.5–8%), and creams and lotions (concentrations of 0.025–0.075%) [5,6].

Camphor (trimethylbicyclo[2.2.1]heptan-2-one) is a natural product isolated from the camphor laurel tree (Cinnamomum camphora). It is known for its antipruritic, analgesic, and counterirritant properties, and is even approved by the Food and Drug Administration (FDA) as an OTC active ingredient as an antitussive, antifungal, external analgesic, anesthetic, skin protectant that can be used in acne treatment, wart removal, and nasal decongestion and as a counterirritant, expectorant, insect repellent, etc. [7]. It activates similar receptors as capsaicin and menthol. In addition to TRPV1, camphor activates and desensitizes TRPV3 and TRPA1 receptors. Although its actions require higher concentrations than capsaicin, camphor acts more rapidly and completely [8]. The literature states that camphor was used in ancient China, India, and Persia (Iran) as an herbal medicine for the treatment of inflammation-related diseases, such as rheumatism, indigestion, bronchitis, asthma, and muscle pain. It is commonly used in the forms of creams, vapour rubs, gels, and balms. Due to its insect-repellent properties against mosquitos, it is suggested as an alternative green insect repellent to synthetic agents that are currently in use [9,10].

Propolis is a natural bee product with extensive biological properties [11]. It is a resinous material, the major bioactive components of which are polyphenols, including phenolic acids and flavonoids [11,12]. Propolis is soluble in ethanol and propylene glycol, which allows for the removal of resins and wax while keeping all the bioactive components intact [13]. Poplar-type propolis is common in Europe and is characterized by a high content of flavonoids. The most abundant polyphenols of poplar-type propolis are pinocembrin, galangin, chrysin, and ρ-coumaric acid [11,14].

When attempting to expand our business to the US market, we contacted US Registrar Corp. They reviewed our labeling and product ingredients and subsequently gave recommendations for adjustment for the US market. The simplest thing was to adjust our traditional product to match the tentative monograph for external analgesic OTC, where camphor and capsaicin are identified as active ingredients. The tentative monograph for external analgesic OTC requires a 0.03% concentration of capsaicin and 3.16% concentration of camphor. Implementation of these requirements is controlled by the FDA (Food and Drug Administration). Considering our traditional product already contained capsaicin and camphor but in minor concentrations, our task was to produce a product with a higher concentration of active ingredients without altering its homogeneity. Determining the quantitative content of active ingredients in the ointment as a finished product was difficult because it also contains a tincture of poplar-type propolis (resins of propolis originate from poplar trees, lat. *Populus* spp.), in which camphor is dissolved. Flavonoids from propolis interfered with the quantification of camphor in the ointment since they a similar retention time as flavonoids from poplar-type propolis.

Reviewing the literature, it was noticed that various methods for capsaicinoid determination have been performed, including liquid chromatography [15,16,17,18,19,20], which was the most used technique; gas chromatography [21,22]; gas chromatography-mass spectrometry FID [23]; a colorimetric method [24]; and spectrophotometric methods [25,26]. Methods used for the determination of camphor were less diverse and were mostly performed with gas chromatography [27,28,29] and HPLC [30]. Propolis analyses were mostly performed with liquid chromatography [31,32]. Other techniques have also been used, such as capillary gas chromatography [33], gas chromatography-mass spectrometry [34], etc. No method for simultaneous determination of the analytes of interest was found. It was therefore necessary to develop a method that, after extraction, could reliably and accurately quantify capsaicin and camphor in a pharmaceutical form, which, according to US legislation, is classified as an over-the-counter (OTC) medicine.

## 2. Results

### 2.1. Method Development

Existing methods were not applicable for the determination of capsaicinoids and camphor in a complexed matrix (ointment) containing propolis. Due to this problem, we developed a method that enables simultaneous analysis of capsaicinoids and camphor. The idea of simultaneous determination of both groups of analytes came from the lack of analytical methods for camphor determination that were applicable in our laboratory. The ointment that was analyzed is a non-prescription drug with a declared amount of capsaicin (0.03%) and camphor (3.16%). The analyzed ointment was developed from a traditional ointment intended to relieve pain and inflammation that accompanies rheumatic diseases. The ointment contained poplar-type propolis, in which camphor was dissolved. Due to the complexity of samples and the similar retention times that polyphenols showed during the analysis, the running time of the method had to be prolonged so that all the polyphenols could be separated from capsaicinoids and camphor for proper determination. To accomplish this, we used a C18 HPLC column similar to the one that we use for the determination of polyphenols. Camphor is a very volatile compound; thus, the temperature of the column and oven should not exceed 25 °C. The mobile phase used in this analysis contained acetonitrile and ultrapure water in a ratio of 2:3 and was adjusted to pH 3.2 using glacial acetic acid. This pH showed the best results in the separation of all analytes in our complex matrix. To ensure that propolis did not affect the concentration of the analytes of interest, extensive matrix-effect analyses were conducted, and results were compared. Compared data obtained results from the analysis of the matrix of the ointment (vaseline + lanolin) alone, a matrix with a known amount of propolis, a matrix with a known amount of standards of capsaicinoids and camphor, a matrix with a known amount of capsicum oleoresin, and model of the ointment made in the laboratory. Subsequently, the results showed that propolis does not affect the determination or quantification of either of the analytes of interest. Chromatograms of these analyses are presented in the Supplementary Materials (Figures S1–S5).

#### Optimization of Extraction of Capsaicinoids and Camphor

Multiple extractions of capsaicinoids and camphor were done by heating the sample ointment in a water bath and ultrasound water bath, with different heating times, starting at 150 min, 120 min, 90 min, and 60 min and finishing after 30 min. Different temperatures were also included, starting at 80 °C and 65 °C and finishing at 50 °C. The results did not show any differences between chosen temperature, so extraction at 50 °C for half an hour was chosen as the fastest method. Extraction was performed with absolute ethanol and 96% ethanol, but no difference in results was shown. Extraction of capsaicinoids from oleoresin was also done with different solvents. Both absolute and 96% ethanol were tested, and acetone and acetonitrile were also compared. Given the fact that there were no differences between absolute and 96% ethanol and between acetone and acetonitrile, 96% ethanol and acetonitrile were chosen as the cheapest and most accessible solutions. Optimization data are not presented in this paper.

The efficiency of the extractions with the chosen solvents was examined by comparing the mean values of the obtained data from the analysis of the finished product and the finished product to which a precisely determined amount of analytes were added. Recovery was calculated and is shown in Table 1. All analyses were performed in triplicate.

### 2.2. Method Validation

The developed HPLC method was adjusted to fit the samples for analysis and the device on which it was applied. The method was validated for system suitability, selectivity, calibration range, accuracy, repeatability, and intermediate precision of measurement of the peak area following the current ICH guidelines [35].

**Figure 2:**
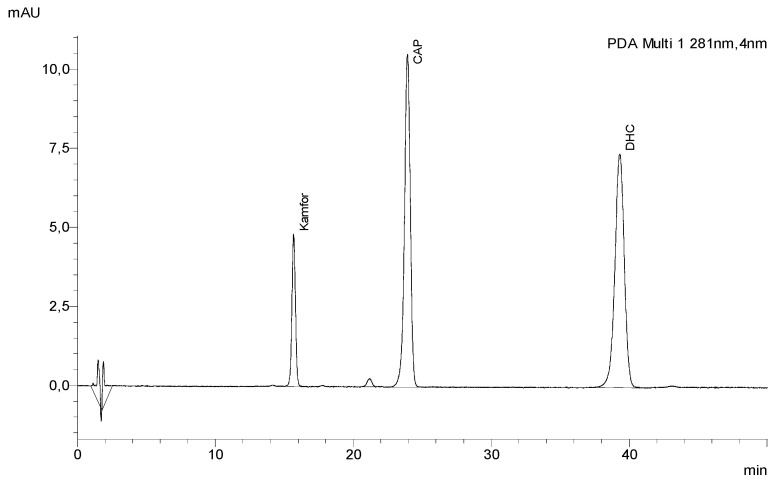
Chromatogram of standard solutions of capsaicin (CAP) (100 µg/mL), dihydrocapsaicin (DHC) (100 µg/mL), and camphor (Kamfor) (1000 µg/mL).

The selectivity of the method was studied by measurement of peak purity using LabSolution software. Evaluation of peak purity was performed to ensure that no comigration substance contributed to the response of the peaks. The linearity range was evaluated by initiating a series of standard solutions (Figure 2) at different concentration levels: 2.5–200 µg/mL for capsaicin and dihydrocapsaicin and 25–2000 µg/mL for camphor. Linear concentration range, LOD, LOQ, and other parameters are summarized in Table 2. The limit of detection (LOD) and the limit of quantification (LOQ) were calculated based on the standard deviation of the response and the slope following the equations:

LOD = 3 Q/S

LOQ = 10 Q/S

where Q is the standard deviation of the response, and S is the slope of the calibration curve. The obtained results show a high sensitivity of the developed method, which allows for its application in a pharmaceutical form of our interest.

Precision was calculated from the data of repeatability (intraday) and intermediate (interday) precision. These measurements were performed by initiating a mixture of standards (capsaicin and dihydrocapsaicin, 100 µg/mL; camphor, 1000 µg/mL) to assess repeatability and intermediate precision. Intraday precision was determined by multiple injections (*n* = 5) of the standard solutions to determine variations in peak area and retention times. Low RSD values show that the repeatability of the method is acceptable (Table 3). Interday precision was evaluated over 3 days. Each day, newly prepared standard solutions were used. Multiple injections of these solutions (*n* = 5) on each day were evaluated. RSD values of retention times and peak areas for capsaicin, dihydrocapsaicin, and camphor indicate that the intermediate precision is acceptable.

The accuracy of the method was determined by performing a recovery study by adding known values of the standard solution to the ointment samples. All analyses were performed in triplicate. Capsaicin and camphor were added in double the amount of that declared by the producer, and dihydrocapsaicin was added at a rate of 50% of the declared amount of capsaicin. This, smaller amount of dihydrocapsaicin was taken to fit a similar amount of dihydrocapsaicin in chili pepper oleoresin. Recovery values, presented in Table 1, show that the accuracy of the method was good. In robustness testing, wavelength changes were made. Analyses were performed in triplicate at one concentration level, with wavelengths of 279, 281, and 283 nm. The obtained values were 48.64 µg/mL ± 1.37% for capsaicin, 48.99 µg/mL ± 1.27% for dihydrocapsaicin, and 508.87 µg/mL ± 0.80%, which indicates that the method was found to be robust.

### 2.3. Application

In order to test the applicability of this newly developed HPLC method, 11 commercially available products containing capsaicinoids, camphor, or both were analyzed (Supplementary Materials Table S1). Analyzed samples were divided into two groups, classified as cosmetics or OTC medications (Table 4). Detailed data about samples can be found in the Supplementary Data (Supplementary Materials Tables S1–S3). Cosmetic products do not have a requirement to specify the amount of active components in the finished product. Subsequently, the method was used for determination and quantification but without the possibility of comparing results with the declaration requirement. Conversely, OTC medications have declaration requirements, and analysis results were compared to them. The applicability of the method was tested by calculating the recovery value of extraction of the selected sample, to which a known amount of standard was added (Table 5). Samples for calculation of recovery of the extraction were selected based on the product matrix (samples 5 and 6 had matrices similar to those of the other products).

**Figure 3:**
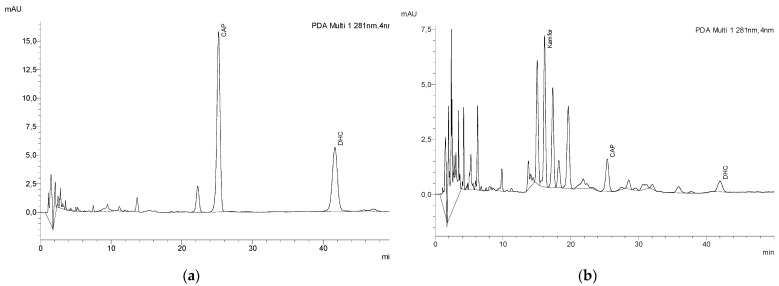
Chromatogram of extracted (**a**) capsicum oleoresin (raw material) and (**b**) finished product.

Our application of the developed method constitutes the first report on the simultaneous determination and quantification of capsaicinoids and camphor by HPLC. Results of the raw materials and finished products show that the method is applicable for different types of samples. Chromatograms (Figure 3a,b) show that this method achieved good separation of all analytes, which is the main advantage of this method. Although the method has a long duration, it is important to emphasize that this long run is what makes the separation of all flavonoids from propolis and our analytes of interest possible. This analytical method does not require expensive equipment, high chemical waste, or complicated sample preparation. It is easily applicable in many laboratories that own HPLC systems. It allows for the determination and quantification of both capsaicinoids and camphor in a single run.

## 3. Discussion

The purpose of this study was to develop a new method for simultaneous determination of capsaicinoids and camphor in a complex matrix—in our case, ointment containing capsicum oleoresin, camphor, and poplar-type propolis. By reviewing the literature, we found a wide range of papers with methods for determination of each analyte separately. Our main interest was to create a new analytical method to determine all our analytes of interest in a single run with the possibility of avoiding interference by polyphenols of propolis.

The majority of the published methods for the determination of capsaicinoids are based on a quick determination of analytes, with a low calibration curve and depending on samples (*Capsicum frutescens*) with longer extraction times [36]. As for camphor, there are not many HPLC methods that are available. The method described by Shaikh and Jain [30] determines camphor, curcumin, and piperine in samples of ayurvedic dental powder. The authors presented a method with a short run of only 10 min, easy sample preparation, a relatively small calibration range (4–8 µg/ ml), and a mobile phase with a similar pH value. The method presented in this paper has a longer run in order to achieve the necessary separation and selectivity, as well as an additional but simple sample preparation due to the complexity of the sample matrix. The method is applicable for different samples containing capsaicinoids and camphor and, as described earlier, is suitable for different matrices of those samples. The method described by Shaikh and Jain [30] is not applicable for this type (ointment) of pharmaceutical formulation due to its simple extraction model, which would lead to poor separation of camphor and thus low selectivity of the method.

Sharma et al. [36] presented a method for capsaicinoid determination in capsicum oleoresin directly extracted from *Capsicum frutescens*; this method could be only partially acceptable for our laboratory for analysis of raw materials. Both methods [30,36] presented higher LOD and LOQ values for analytes of interest, as well as a small calibration range, and are not applicable to a complex sample, such as pharmaceutical formulations containing different solvents, excipients, and active components, which could interfere with determination and quantification. Another benefit of our proposed method is the determination of dihydrocapsaicin, the presence of which in a ratio of 1:2 with capsaicin suggests that the raw material is a natural product [23]. Coupling HPLC with a mass spectrometer would provide better selectivity and higher sensitivity of the method, which we hope to be the next step in the development of our laboratory.

## 4. Materials and Methods

### 4.1. HPLC Instrumentation and Chromatographic Conditions

Analysis of OTC medications for topical use and chili pepper oleoresins was performed on a Shimadzu HPLC system with LabSolution software, two LC-20ADXR solvent-delivery units, a CTO-20AC column chamber, an SPD-M20A diode-array detector, and a SIL20-ACX autosampler. The separation of components was achieved on a Waters Symmetry^®^ C18 column (4.6 × 250 mm; 5 μm)

### 4.2. Chemicals and Reagents

Capsaicin (purity ≥ 95%), dihydrocapsaicin (purity ≥ 85%), and camphor (≥97.5%) were purchased from Sigma-Aldrich (St. Louis, MI, USA). Acetic acid (glacial) (100%) was obtained from Merck (Darmstadt, Germany). J.T. Baker HPLC-grade acetonitrile was purchased from Avantor (Gliwice, Poland). Ethanol absolute anhydrous (Ph. Eur. reagent) was purchased from Carlo Erba (Barcelona, Spain), and ultra-pure water was obtained with a Letzner water-purification system (Hückeswagen, Germany).

### 4.3. Preparation of Standard Solutions

Capsaicin and dihydrocapsaicin (10 mg of each) were accurately weighed, transferred to 10 mL volumetric flasks, and dissolved in 10 mL of absolute ethanol. Camphor standard (100 mg) was accurately weighed, transferred to 10 mL volumetric flasks, and dissolved in 10 mL of absolute ethanol. From these solutions, 10 aliquots were made and stored at −15 °C with temperature monitoring using a LOG200 data logger purchased from Dostmann Electronic GmbH (Reicholzheim, Germany). Stock solutions were prepared with a mobile phase in the following concentrations: for capsaicin and dihydrocapsaicin, 2.5, 25, 50, 100, and 200 µg/mL; for camphor, 25, 250, 500, 1000, and 2000 µg/mL.

### 4.4. Sample Preparation

#### 4.4.1. Ointment Preparation

For the analysis, 0.5 g of the ointment was accurately weighed in a 10 mL extraction tube, to which 5 mL of ethanol was added. The sample was extracted for 30 min in a water bath at 50 °C with occasional stirring. After cooling the sample, it was centrifuged for 20 min at 3500 rpm. A volume of 0.5 mL of the supernatant was separated and mixed with 0.5 mL of the mobile phase. The sample was vortexed for a short period and then centrifuged for another 5 min at 3500 rpm. The supernatant was filtered through a 0.45 µm nylon filter and initiated into the system.

#### 4.4.2. Oleoresin Preparation

For oleoresin analysis, 1 g of oleoresin was accurately weighed into a 50 mL volumetric flask, to which 5 mL acetonitrile was added. Oleoresin was dissolved in acetonitrile by swirling the flask. Then, five portions of 5 mL of 96% ethanol were added to the flask and swirled well after each addition. The flask was filled up to a mark with 96% ethanol. A volume of 5 mL of this solution was passed through a C18 solid-phase extraction cartridge (which was preconditioned by flushing with 5 mL of 96% ethanol) in a 25 mL volumetric flask. The cartridge was washed 3 times with 5 mL ethanol in the same flask. The solution was then diluted with 96% ethanol up to the mark and mixed. A 1 mL volume of solution was filtered through a 0.45 µm syringe filter into a 1.5 mL glass vial and initiated into the system.

### 4.5. Mobile Phase and HPLC Analysis

The mobile phase containing acetonitrile and ultrapure water in a ratio of 2:3 was adjusted to pH 3.2 using glacial acetic acid. The mobile phase was degassed with a vacuum pump before use. The flow of the mobile phase was 1.5 mL/min at a column temperature of 25 °C, with an injection volume of 10 μL. Spectrum recording was performed in the wavelength range from 190 to 370 nm, whereas detection was performed at a wavelength of 281 nm. The run was 50 min long, with the following relative retention times: capsaicin, 22.9; dihydrocapsaicin, 37.45; and camphor, 15.23 min. Identification of capsaicinoids and camphor was performed by comparing the retention times of standard solutions and sample solutions, and unquestionable identification was confirmed by comparing the specific spectrum of capsaicinoids and camphor standard solutions with that of sample solutions. The detector response was linear over the range of 2.5 to 200 µg/mL for capsaicin and dihydrocapsaicin and the range of 25 to 2000 µg/mL for camphor. It is advisable to purge the HPLC column with 100% acetonitrile after every three to four samples under the same chromatographic conditions during analysis.

## 5. Conclusions

An isocratic HPLC method coupled with a DAD detector for simultaneous determination of capsaicinoid and camphor was developed with the specific aim of determining and quantifying capsaicinoid and camphor content in OTC medications for topical use. This method is also applicable for the determination and quantification of capsaicinoids in capsicum oleoresin, which was used as raw material for the same product. The method ensures a quantitative and reproducible determination of capsaicinoids and camphor and was validated according to the current ICH Q2(R1) guidelines. In conclusion, this analytical method can be used for quality control of formulated pharmaceutical products containing capsaicinoids, camphor, and propolis.

## Figures and Tables

**Table 1:** Recovery values for capsaicin, dihydrocapsaicin, and camphor were calculated at a confidence level of 95% (CI).

Analyte	Recovery Mean (%) ± SD *	RSD (%) *	CI
Capsaicin	97.33 ± 1.95	2.00	1.71
Dihydrocapsaicin	97.17 ± 1.16	1.20	1.02
Camphor	97.71 ± 1.24	1.27	1.09

* SD—standard deviation; RSD—relative standard deviation.

**Table 2:** Validation parameters for capsaicin, dihydrocapsaicin, and camphor quantification.

Analyte	Linearity Range (µg/mL)	Regression Equation	R^2^	LOD (µg/mL)	LOQ (µg/mL)
Capsaicin	2.5–200	y = 3094.41x	0.999	0.070	0.212
Dihydrocapsaicin	2.5–200	y = 3489.10x	0.999	0.211	0.640
Camphor	25–2000	y = 85.8936x	0.999	0.060	0.320

**Table 3:** Precision data for capsaicin, dihydrocapsaicin, and camphor.

Analyte	Intraday Precision (*n* = 5)				Interday Precision (*n* = 5)			
	Retention Time	RSD (%)	Peak Area	RSD (%)	Retention Time	RSD (%)	Peak Area	RSD (%)
Capsaicin	23.30	0.19	321527.60	0.27	23.59	0.45	323533.60	0.43
Dihydrocapsaicin	38.20	0.23	349557.20	0.16	38.17	3.06	351753.40	0.22
Camphor	15.42	0.08	85639.60	0.28	15.52	0.27	85804.80	0.49

**Table 4:** Results of analyzed commercially available products containing analytes of interest, classified by category.

Sample Number	Sample Category	Analyte of Interest	Declaration Requirement (µg/g)	Concentration of Capsaicin (µg/g)	Concentration of Dihydrocapsaicin (µg/g)	Concentration of Camphor (µg/g)
S1	Cosmetics	Camphor	n.s.*	n.c.*	n.c.	94,519.00
S2		Camphor	n.s.	n.c.	n.c.	91,304.50
S3		Camphor	n.s.	n.c.	n.c.	45,840.00
S4		Capsaicinoids	n.s.	68.50	60.00	n.c.
S5		Capsaicinoids/Camphor	n.s.	241.60	131.40	35,547.00
S6		Capsaicinoids/Camphor	n.s.	54.80	18.80	6108.20
S7		Capsaicinoids/Camphor	n.s.	168.20	n.c.	6011.40
S8		Capsaicinoids/Camphor	n.s.	60.60	175.00	2991.40
S9	OTC medication	Capsaicinoids	4000	3619.20	n.c.	n.c.
S10		Capsaicinoids	750	477.80	181.00	n.c.
S11		Capsaicinoids/Camphor	300 / 31,600	299.20	222.00	35,806.80

* n.s.—not specified in the declaration of the sample * n.c.—not contained in the sample.

**Table 5:** Recovery values of extraction of commercially available products.

Sample Number	Recovery (%)	
	Capsaicin (%)	Camphor (%)
S1	n.c.*	103.68
S2	n.c.	101.17
S3	n.c.	102.18
S4	100.45	n.c.
S7	102.29	94.34
S8	99.46	98.07
S9	95.15	n.c.
S10	103.32	n.c.
S11	102.29	94.34
S11	98.57	103.30

* n.c.—not contained in the sample.

## Data Availability

All supporting data can be obtained from the corresponding author upon formal request.

## Supplementary Materials

The following are available online: Figure S1. Chromatogram of matrix (vaselin + lanolin); Figure S2. Chromatogram of matrix (vaselin + lanolin) + propolis; Figure S3. Chromatogram of matrix (vaselin + lanolin) + camphor; Figure S4. Chromatogram of matrix (vaselin + lanolin) + Chilli pepper Oleoresin; Figure S5. Chromatogram of standard solution of capsaicinoids (50 µg/mL CAP, 50 µg/mL DHC) and camphor (500 µg/mL); Figure S6. Chromatogram of the model of the ointment; Table S1. List of analyzed products with ingredients listed as labeled; Table S2. Content of analytes of interest; Table S3. Extraction recovery of the analyzed products.

## Sample Availability

Samples of the compounds are available from the authors.
