# Supplementary material for: Development and Validation of an HPLC Method for Simultaneous Determination of Capsaicinoids and Camphor in Over-the-Counter Medication for Topical Use
Source: Molecules. 2022 Feb 14;27(4):1261. doi: 10.3390/molecules27041261 (PMC8878854; doi:10.3390/molecules27041261)
Supplement: Supplementary file 1 [file molecules-27-01261-s001.zip › molecules-1583618-supplementary.pdf]

Table S1. List of analyzed products with ingredients listed as labeled

| Finished product | Analyt of interest | Ingrediants |
|---|---|---|
| Apipharma, Kapsin ointment | capsaicinoids / camphor | **Active components: Capsicum oleoresin (300 µg /g ointment), Camphor (31600 µg /g ointment)**, Inactive ingrediants: Petrolatum, Lanolin Cera, Alcohol, Aqua, Propolis Cera |
| Apipharma, Kapsin ointment | capsaicinoids / camphor | Petrolatum, Lanolin Cera, Alcohol, Aqua, Propolis Cera, **Capsicum extract, Camphor** |
| Apipharma, Propireumin | camphor | Petrolatum, Lanolin Cera, Alcohol, Aqua, Propolis Cera, Menthol, **Camphor** |
| Biovitalis, Paprika gel | capsaicinoids / camphor | Aqua, glycerin, **camphor**, methyl salicylate, **capsicum frutescens resin**, rosmarinus officinalis leaf oil, gaultheria procumbens leaf oil, sodium hydroxide, carbomer, benzyl alcohol, dehydroacetic acid, CI 16255, limonene, linalool |
| Dr. Theiss, Allga San Mobil Creme | capsaicinoids / camphor | Aqua, Alcohol Denat., Cetearyl Alcohol, Glyceryl Stearate Citrate, Sucrose Stearate, Hydrogenated Polydecene, Dicaprylyl Ether, Simmondsia Chinensis Seed Oil, Ricinus Communis Seed Oil, Pentylene Glycol, Glycerin, Panthenol, Tocopheryl Acetate, Vanillyl Butyl Ether, Phenoxyethanol, Symphytum Officinale Extract, Rosmarinus Officinalis Leaf Oil, Hydrogenated Castor Oil, Pinus Mugo Leaf Oil, **Camphor**, Parfum, Carbomer, Allantoin, Sodium Hydroxide, Ethylhexylglycerin, Benzyl Nicotinate, **Capsicum Frutescens Oleoresin,** Helianthus Annuus Seed Oil, Limonene, Linalool |
| Dr. Theiss, Allga San Mobil Fluid | capsaicinoids | Aqua, Alcohol Denat., PEG-60 Hydrogenated Castor Oil, Vanillyl Buthyl Ether, Symphytum Officinale Leaf Extract, Panthenol, Isobornylacetat, Propylene Glycol, Parfum, Pinus Mugo Leaf Oil, Rosmarinus Officinalis Oil, Arcina Montana Flower Extract, Allantoin, Salvia Lavandulaefolia Leaf Oil, Rosmarinus Officinalis Leaf Oil, Benzyl Nicotinate, **Capsicum Frutescens Oleoresin**, Potassium sorbate, Citric acid, Limonene, Linalool |
| Esensa, Khan, tiger balm | camphor | Petrolatum, Paraffinum Liquidum, **Camphor**, Menthol, Eucaliptus Globulus Oil, Cera Alba, Eugenia Caryophyllus Oil |
| Esensa, Khan, tiger cream gel | camphor | Aqua, **Camphor**, Menthol, Isopropyl Myristate, Eucalyptus Globulus Leaf Oil, Glycerin, Propylene Glycol, Methyl Salicylate, Polysorbate 20, Triethanolamine, Carbomer, Phenoxyethanol, Eugenia Caryophyllus Leaf Oil, Ethylhexylglycerin |
| Hamapharm, FlexoMax | capsaicinoids / camphor | Aqua, Alcohol Denat, **Capsicum Frutescens Fruit extract**, Salix Alba Bark Extract, Harpagophytum Procumbens Root Extract, Curcuma Xanthorrhiza Root Extract, Menthol, Methyl Salicylate, Sodium Hydroxide, **Camphor**, Glucosamine Sulfate, Acrylates/C10-30 Alkyl Acrylate Crosspolymer, Benzyl Alcohol, Ethylhexylglycerin, Tocopherol |
| Hansaplast, ABC, warme-creme | capsaicinoids | **Active ingrediant: Capsicum (750 µg/g ointment),** Inactive ingrediants: methyl parahydroxybenzoate (E218), propyl parahydroxybenzoate (E216), macrogrolcetyl stearyl ether, cetyl stearyl alcohol, isopropyl myristate, viscose paraffin, propylene glycol, sodium citrate, citric acid monohydrate, purified water, sodium hydroxide |
| Sanofi, Finalgon - Salbe | capsaicinoids | **Active ingrediant: Nonylvanillamide (4000 µg/g ointment)** Inactive ingrediants: nicotinic acid-B-butoxyethyl ester, Sorbic acid, citronellol, Diisopropyl adipate, colloidal silicon, white Vaseline, purified water |

Table S2. Content of analytes of interest

| Sample name | Analyt of interest | Content of capsaicin (µg/g) | Content of dihydrocapsaicin (µg/g) | Content of camphor (µg/g) |
|---|---|---|---|---|
| S1 | camphor | n.c.* | n.c. | 94519.00 |
| S2 | camphor | n.c. | n.c. | 91304.50 |
| S3 | camphor | n.c. | n.c. | 45840.00 |
| S4 | capsaicinoids | 68.50 | 60.00 | n.c. |
| S5 | capsaicinoids / camphor | 241.60 | 131.40 | 35547.00 |
| S6 | capsaicinoids / camphor | 54.80 | 18.80 | 6108.20 |
| S7 | capsaicinoids / camphor | 168.20 | n.c. | 6011.40 |
| S8 | capsaicinoids / camphor | 60.60 | 175.00 | 2991.40 |
| S9 | capsaicinoids | 477.80 | 181.00 | n.c. |
| S10 | capsaicinoids | 3619.20 | n.c. | n.c. |
| S11 | capsaicinoids / camphor | 299.20 | 222.00 | 35806.80 |

* n.c.- not contained in the sample

Table S3. Extraction recovery of the analyzed products

| Sample name | Analyt of interest | Content of capsaicin (µg/ml) | Content of capsaicin + STD Capsaicin (µg/ml) | Volume (µl) of an added standard of capsaicin ( 1 mg/ml) | Recovery of capsaicin (%) |
|---|---|---|---|---|---|
| S4 | capsaicinoids | 68.50 | 169.25 | 100.00 | 100.45 |
| S7 | capsaicinoids / camphor | 168.20 | 223.20 | 50.00 | 102.29 |
| S8 | capsaicinoids / camphor | 60.60 | 110.00 | 50.00 | 99.46 |
| S9 | capsaicinoids | 477.80 | 549.80 | 100.00 | 95.15 |
| S10 | capsaicinoids | 3619.20 | 3842.80 | 100.00 | 103.32 |
| S11 | capsaicinoids / camphor | 299.20 | 344.20 | 50.00 | 98.57 |

| Sample name | Analyt of interest | Content of camphor (µg/ml) | Content of camphor + STD Camphor (µg/ml) | Volume (µl) of an added standard of camphor (10 mg/ml) | Recovery of camphor (%) |
|---|---|---|---|---|---|
| S1 | camphor | 94519.00 | 99036.00 | 1000.00 | 103.68 |
| S2 | camphor | 91304.50 | 93384.50 | 1000.00 | 101.17 |
| S3 | camphor | 45840.00 | 47860.00 | 1000.00 | 102.18 |
| S7 | capsaicinoids / camphor | 6011.40 | 6142.80 | 500.00 | 94.34 |
| S8 | capsaicinoids / camphor | 2991.40 | 3424.00 | 500.00 | 98.07 |
| S11 | capsaicinoids / camphor | 35806.80 | 38164.60 | 500.00 | 103.30 |

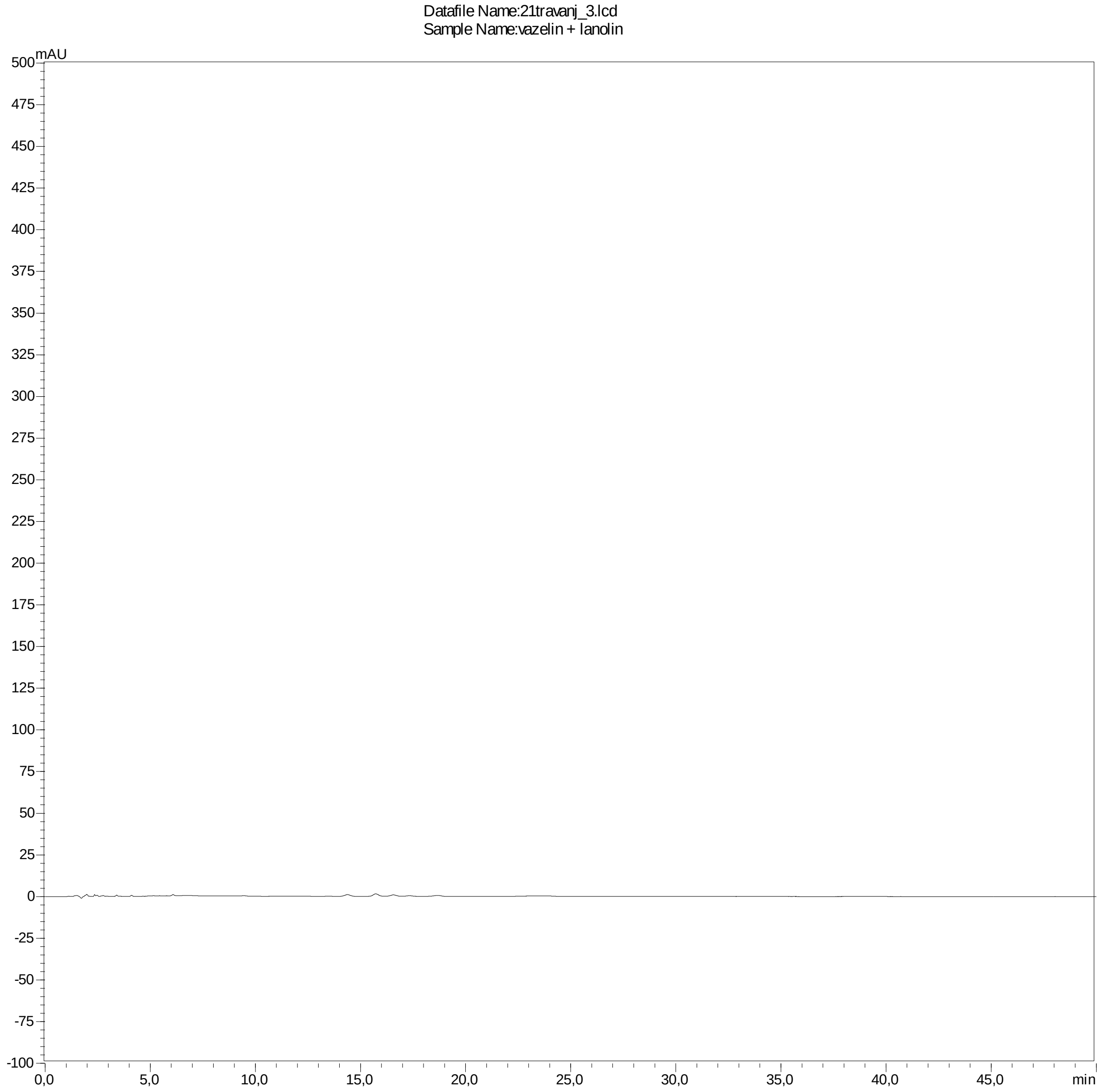


Figure S1. Chromatogram of matrix (vaselin + lanolin)

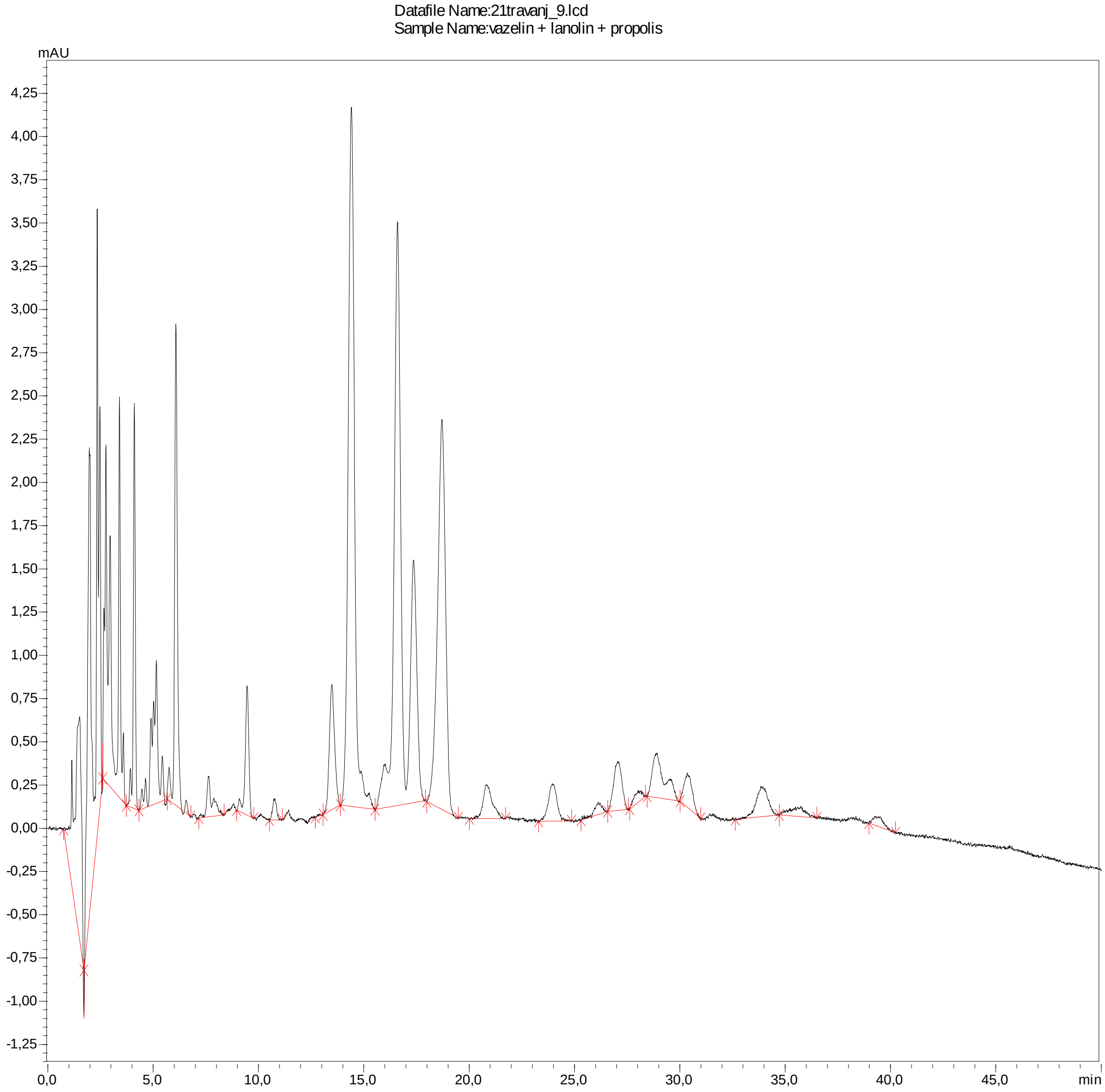


Figure S2. Chromatogram of matrix (vaselin + lanolin) + propolis

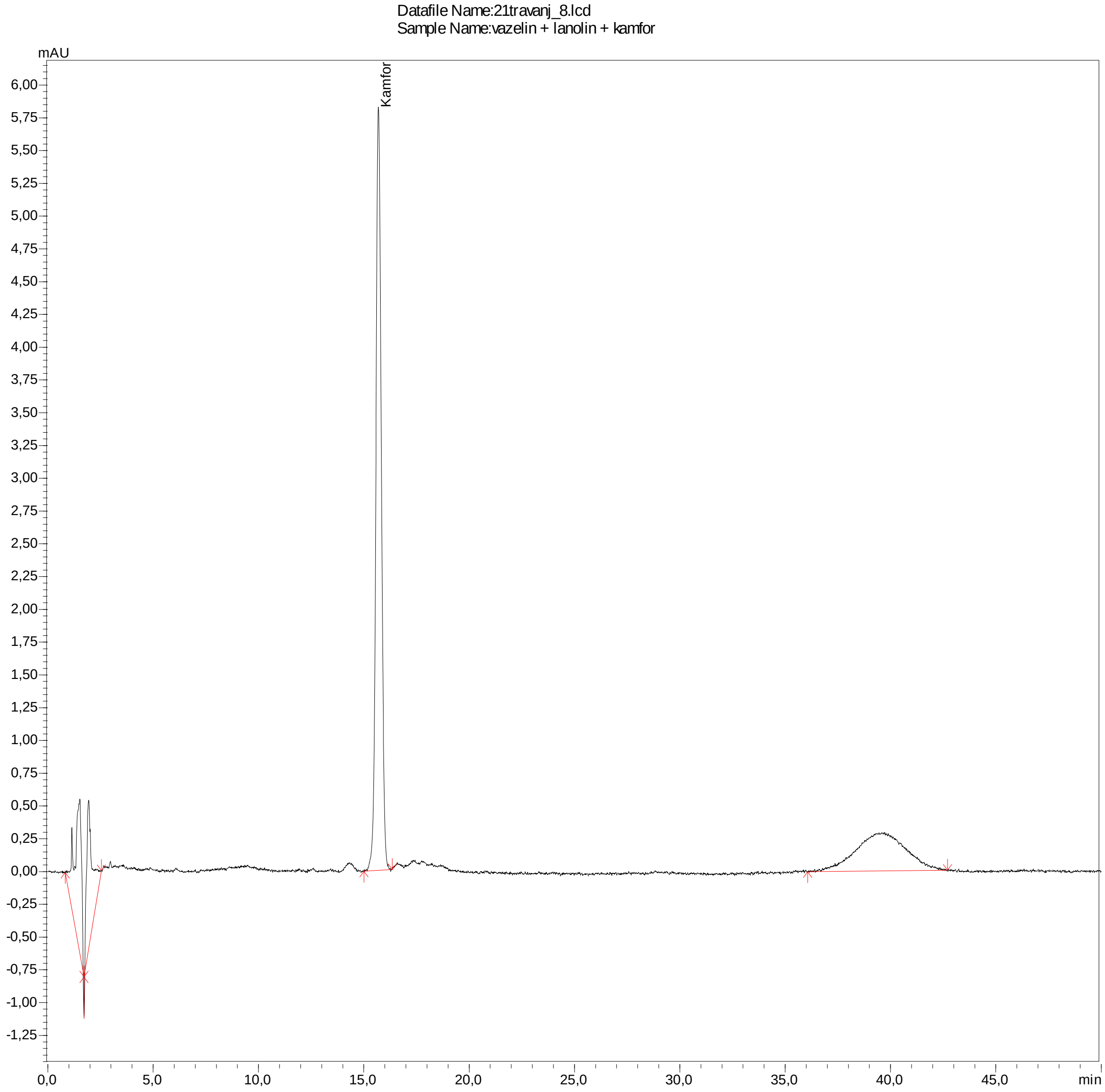


Figure S3. Chromatogram of matrix (vaselin + lanolin) + camphor

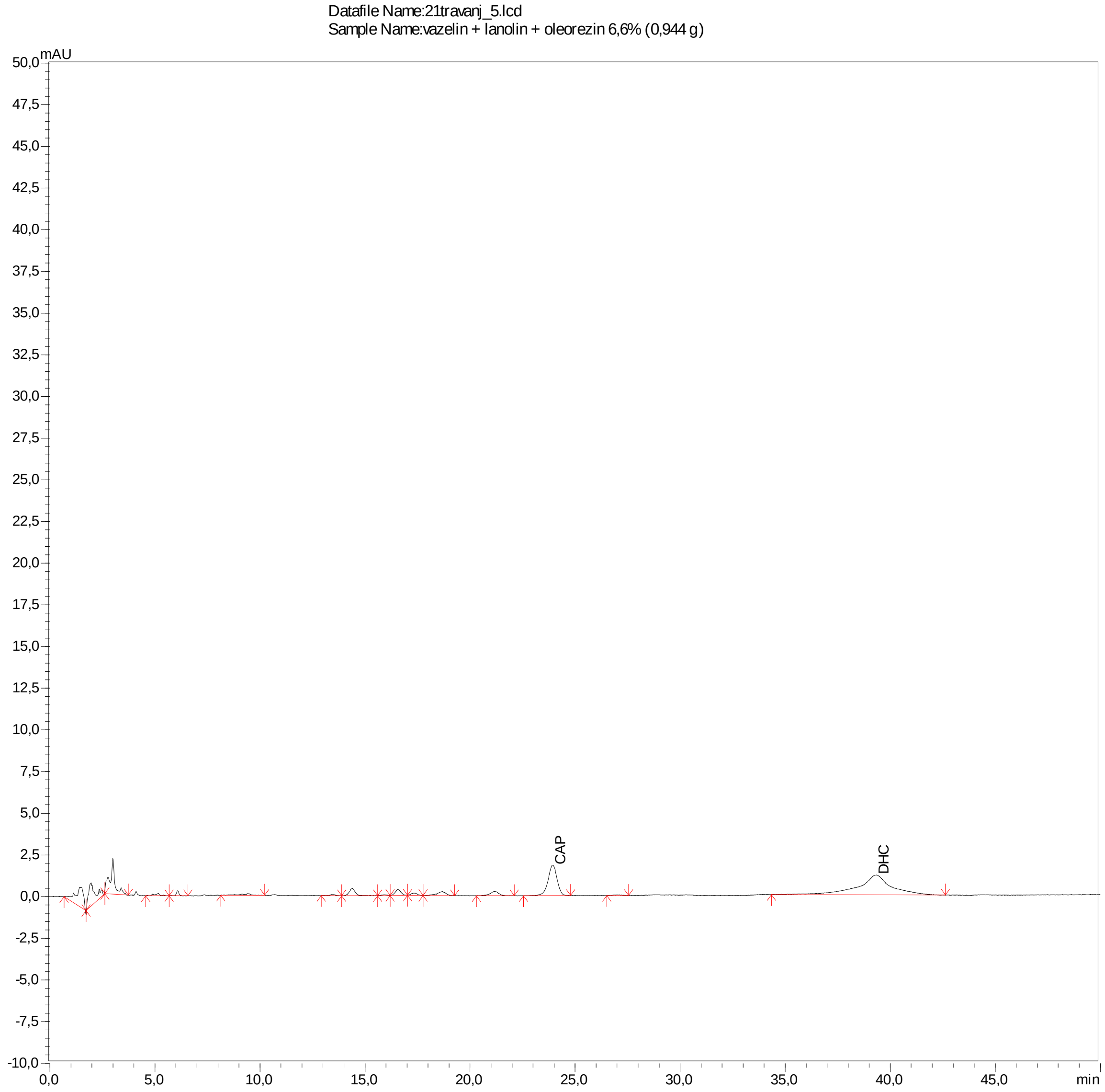


Figure S4. Chromatogram of matrix (vaselin + lanolin) + Chilli pepper Oleoresin

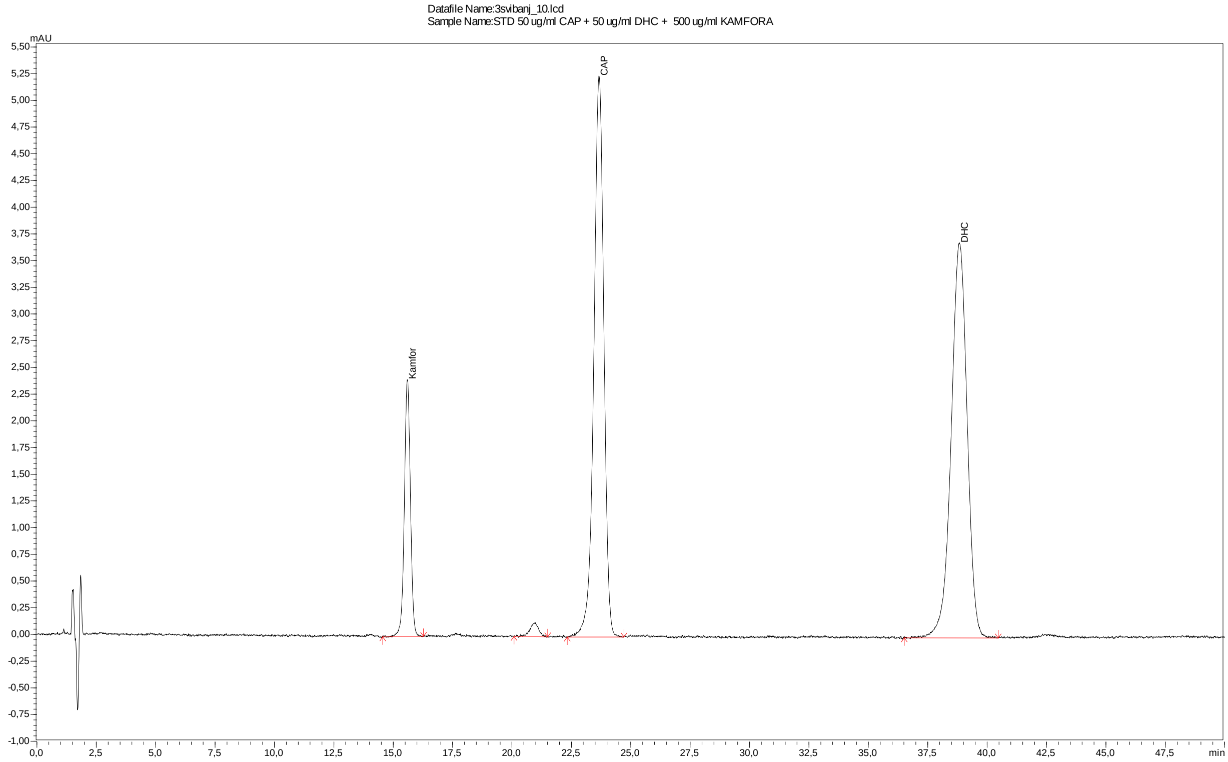


Figure S5. Chromatogram of standard solution of capsaicinoids (50 µg/mL CAP, 50 µg/mL DHC) and camphor (500 µg/mL)

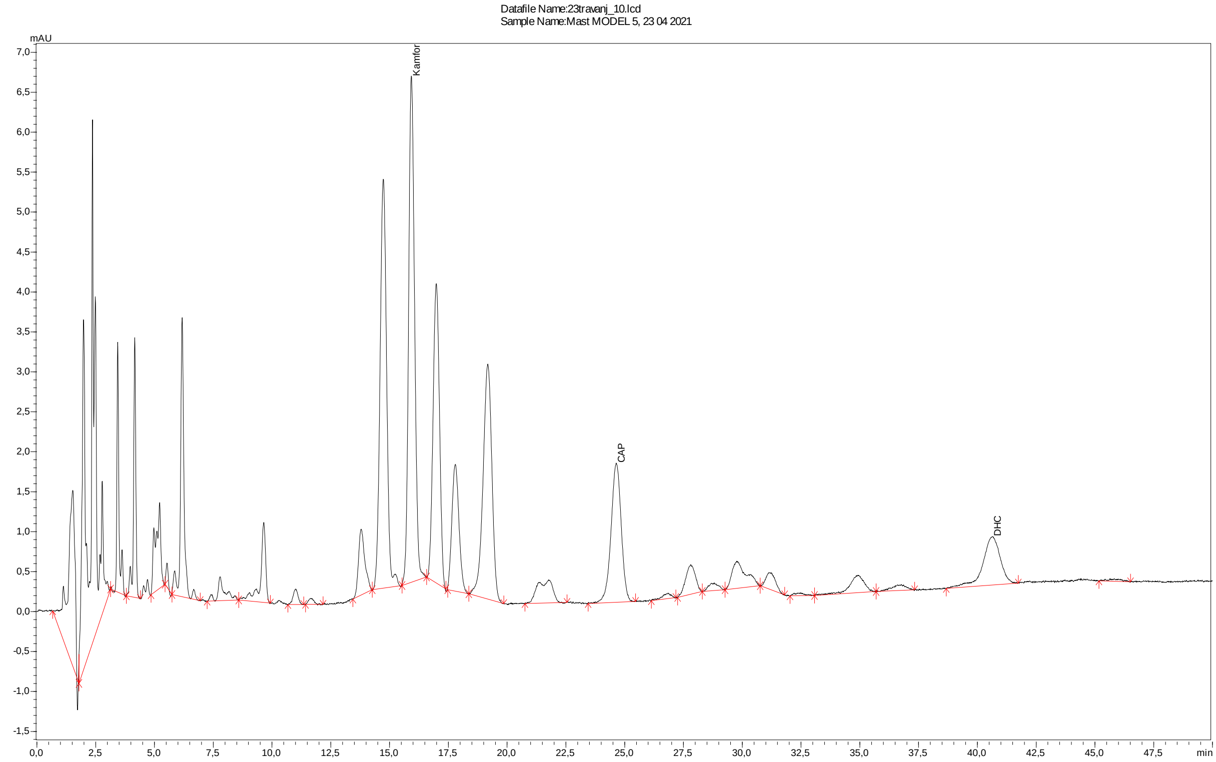


Figure S6. Chromatogram of the model of the ointment
